# Exploring the therapeutic mechanisms of Coptidis Rhizoma in gastric precancerous lesions: a network pharmacology approach

**DOI:** 10.1007/s12672-024-01070-5

**Published:** 2024-06-05

**Authors:** Xuxing Ye, Chao Yang, Hanzhi Xu, Qin He, Lin Sheng, Junmei Lin, Xiaobo Wang

**Affiliations:** 1 Department of Traditional Chinese Medicine, Jinhua Municipal Central Hospital, 351 Mingyue Street, Wucheng District, Jinhua, 321000 Zhejiang China; 2 The Fourth School of Clinical Medicine, Zhejiang Chinese Medical University, 548 Binwen Road, Binjiang District, Hangzhou, 310000 China; 3 Zhejiang University School of Medicine, Hangzhou, 310000 China; 4 Department of Pulmonary and Critical Care Medicine, Jinhua Municipal Central Hospital, Jinhua, 321000 Zhejiang China

**Keywords:** Coptidis Rhizoma, Gastric precancerous lesions, Network pharmacology, Molecular docking, Molecular mechanism, Traditional Chinese Medicine

## Abstract

**Background:**

Gastric precancerous lesions are a critical stage in the development of gastric cancer or gastric adenocarcinoma, and their outcome plays an important role in the malignant progression of gastric cancer. Coptidis Rhizoma has a good effect on Gastric precancerous lesions. However, the specific mechanisms of its action remain incompletely elucidated.

**Methods:**

Network pharmacology and molecular docking techniques were used to explore the active ingredients and molecular mechanism of Coptidis Rhizoma in treating gastric precancerous lesions. The active compounds of Coptidis Rhizoma and their potential gastric precancerous lesions related targets were obtained from TCMSP, GeneCards, and OMIM databases. An interaction network based on protein–protein interactions (PPIs) was constructed to visualize the interactions between hub genes. Analysis of GO enrichment and KEGG pathway were conducted using the DAVID database. An investigation of interactions between active compounds and potential targets was carried out by molecular docking. Finally, animal experiments were conducted to verify the effect and mechanism of Coptidis Rhizoma in treating precancerous lesions of gastric cancer.

**Results:**

A total of 11 active compounds and 95 anti-gastric precancerous lesions targets of Coptidis Rhizoma were screened for analysis. GO enrichment analysis showed that the mechanism of Coptidis Rhizoma acting on gastric precancerous lesions involves gene expression regulation and apoptosis regulation. KEGG pathway enrichment analysis showed that Coptidis Rhizoma against gastric precancerous lesions involving the AKT /HIF-1α/VEGF signalling pathway. Molecular docking simulations indicated potential interactions between these compounds and core targets involved in anti-gastric precancerous lesions activity. In addition, it was confirmed in vivo that Berberine and Coptidis Rhizoma may reverse atrophy and potential intestinal metaplasia by inhibiting the expression of p-AKT, HIFA, and VEGF.

**Conclusion:**

Bioactive compounds in Coptidis Rhizoma have the potential to prevent atrophy and intestinal metaplasia. These compounds function by regulating the proteins implicated in AKT /HIF-1α/VEGF signalling pathways that are crucial in gastric epithelial cell differentiation, proliferation and maturation.

**Supplementary Information:**

The online version contains supplementary material available at 10.1007/s12672-024-01070-5.

## Introduction

Gastric cancer is the fourth most common cancer worldwide, with high morbidity and mortality [1]. Most gastric cancers are thought to develop from gastric precancerous lesions (GPL), including chronic atrophic gastritis (CAG) and intestinal metaplasia (IM) [2]. GPL refer to lesions with histomorphological abnormalities and the risk of malignant transformation of gastric cancer [3], which are common diseases of the digestive system and often occur in the gastric mucosa, accompanied mainly by gastric epithelial dysplasia (GED) and IM [4]. GPL are a critical stage in the development of gastric cancer or gastric adenocarcinoma, and their outcome plays an important role in the malignant progression of gastric cancer [5]. Modern studies suggest that the mechanism may be cross-talk between the development of precancerous lesions and oncogenic signalling pathways in the progression of gastric cancer resulting in the imbalance in the proliferation and apoptosis of gastric epithelial cells [6, 7]. Therefore, early intervention of precancerous lesions is an effective strategy to prevent the development of gastric cancer [8]. Endoscopic mucosal resection (EMR) and endoscopic submucosal dissection (ESD) have emerged as important means of treating GPL [9], but they also have the disadvantage of relatively high local recurrence rates and metachronous lesions, and histologically incurable may arise when lesions have submucosal invasion [10].

Traditional Chinese Medicine (TCM) has been inherited in China for thousands of years because of its reliable efficacy. Modern studies suggest that TCM performs remarkably in cancer prevention, immune regulation, and tumor microenvironment [11–13]. Many TCM famous prescriptions, single-drug prescriptions and proven prescriptions are widely used in treating various diseases, including digestive system diseases, such as GPL and gastric cancer. According to TCM theory, the main pathogenesis of GPL is dampness-heat, turbidity poison [14]. Coptidis Rhizoma is a famous Chinese herbal medicine, which has the effects of clearing away heat and dampness, purging fire and detoxifying, it can regulate immunity, inhibit tumors and control inflammation [15], and is often used in the treatment of the digestive system diseases and malignant tumors [16, 17]. In addition, Coptidis Rhizoma has various effects, such as neuroprotection, anti-oxygen, anti-atherosclerosis, anti-diabetes, and anti-obesity [15]. At present, several studies have confirmed that berberine (BBR) and its derivatives, the active components of Coptidis Rhizoma, have a good effect in inducing tumor cell apoptosis [18, 19]. However, its specific mechanism of action remains incompletely elucidated.

**Fig. 1:**
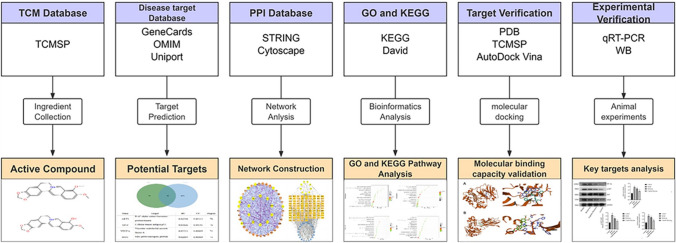
Workflow of the network pharmacological investigation on Coptidis Rhizomain in the treatment of gastric precancerous lesions

The occurrence and development of tumors are associated with abnormal signalling networks formed by cellular molecular pathways [20]. This is also true for GPL. Therefore, attempts to suppress tumors through a single pathway tend to be less effective. Complex signalling networks can often promote bypass mechanisms to confer tumor resistance [21]. Chinese herbal medicines have the advantages of “multi-component” and “multi-target” [22], and it is the current trend to find single drugs or natural compounds acting on multi-target, multi-pathway, and multi-directional regulation of cancer therapy [23]. In this study, network pharmacology and molecular docking techniques were used to explore the active ingredients and molecular mechanism of Coptidis Rhizoma in treating GPL (Fig. 1).

## Materials and methods

### Acquisition of active ingredients

The acquisition of active ingredients of TCM came from the Traditional Chinese Medicine Systems Pharmacology (TCMSP, https://tcmsp-e.com/tcmsp.php). TCMSP is a database containing Chinese herbal medicines, ingredients with structural files, and ADME attributes that focus on exploring the associations between active ingredients, targets of action, and diseases of Chinese herbs [24]. Oral bioavailability (OB) ≥ 30% and drug-likeness (DL) ≥ 0.18 were used as screening criteria to obtain pharmacodynamic components.

### Coptidis Rhizoma target mining

The collected effective active ingredients were used to obtain the corresponding targets in the TCMSP database and integrated to obtain the drug targets, and then UniProt (https://www.uniprot.org/), the database was processed for target matching and gene name normalization for network structure analysis.

### Disease target screening for GPL

Using the GeneCards database (https://www.genecards.org/) and Online Mendelian Inheritance in Man database (OMIM, https://www.omim.org/) yielded disease-related targets for GPL. Using Draw Venn Diagram online tools (http://bioinformatics.psb.ugent.be/webtools/Venn/), extract the intersection of the Coptidis Rhizoma target and GPL disease target, which is the potential target of Coptidis Rhizoma in the treatment of the GPL.

### PPI (protein–protein interaction) network construction

The potential targets obtained from the above screening were imported into the STRING database (https://string-db.org/), and protein interaction network data information was generated online (high: > 0.7; medium: > 0.4; low: > 0.15). The PPI network diagram was then constructed using Cytoscape 3.8.2 software. Key modules were screened using the MCODE plug-in, and hub gene screening was performed using cytohubba.

### GO and KEGG enrichment analysis

Potential targets screened were uploaded into the David 6.8 database (https://david.ncifcrf.gov/), gene ontology and pathway enrichment analysis of targets were performed to screen the top 20 entries with P ≤ 0.01, respectively, and data visualisation was performed. Then the screened active ingredients, targets and pathways data were collated to obtain the corresponding data information and imported into Cytoscape 3.8.2 software to generate the “component–target–pathway” network diagram.

### Molecular docking

The molecular formula of the active ingredient of Coptidis Rhizoma was downloaded from the TCMSP database as a ligand and from the PDB database (https://www.rcsb.org/), key target proteins were downloaded, and water molecules and ligands were removed as receptors. Batch molecular docking of the selected ligands and receptors was performed one by one to verify the binding ability using AutoDock Vina 1.2.3 software.

### Animal experiments

#### Materials

30 SPF grade Wistar rats, weighing between 220 and 250 g and aged 4–6 weeks, were procured from the Experimental Animal Center of Lanzhou University. The rats were housed in a controlled environment with a 12-h light–dark cycle, temperature maintained at 23 ± 2 °C, and humidity ranging from 45 to 65%. Ethical approval for this study was obtained from the Animal Ethics Committee of Jinhua Central Hospital under approval number AL-JHYY202330. This study was conducted in strict accordance with ‘Guiding Opinions on Treating Experimental Animals’ by the Ministry of Science and Technology of the People’s Republic of China and principles of the Basel Declaration.

The reagents used in this study include MNNG (batch number: NH8JH-QD, Tokyo Chemical Industry Co., Ltd., Japan), the BCA reagent kit from Beyotime Biotechnology Co., Ltd (Shanghai, China), rabbit anti-vascular endothelial growth factor (VEGF) monoclonal antibody from Santa Cruz Company (United States), rabbit anti-HIF-a antibody from CST Company (United States), phosphorylated AKT (Ser473) (D9E) XP recombinant rabbit monoclonal antibody, AKT (pan) (C67E7) recombinant rabbit monoclonal antibody, and GAPDH rabbit polyclonal antibody from ServiceBio Biotechnology Company (Wuhan, China).The horseradish enzyme-conjugated antibodies, the goat anti-rabbit IgG (batch number ZB-2301) and goat anti-mouse IgG (batch number ZB-2305), were procured from Beijing Zhong Shan-Golden Bridge Biological Technology Co., Ltd. The ECL reagent kit utilized in this study was sourced from Millipore Company (United States).

#### Drug preparation

Coptidis Rhizoma decoction pieces (batch number: 230401501) sourced from Kangmei Pharmaceutical Co., Ltd were utilized in the preparation of Rhizoma Coptidis decoction. A quantity of 50 g of Rhizoma Coptidis decoction pieces was weighed and soaked in 500 mL of pure water for 30 min. The mixture was then heated and brought to a slight boil, followed by a 2-h decoction period. The resulting solution was filtered using gauze, transferred to a rotary evaporator, and concentrated to 250 mL at 90 °C. The solution was then diluted to achieve an equivalent concentration of 0.20 g/mL of the original medicine, resulting in the preparation of Rhizoma Coptidis decoction. The dosage of gavage administration was 10 mL/kg (2.00 g/kg). Berberine hydrochloride (batch number: 1807) was also included in the study.

#### Modeling grouping and administration

The rats were weighed and randomly divided into groups after 1 week of adaptive feeding. The control group (n = 6) received a normal diet and drinking water, along with an equal volume of physiological saline by gavage during administration. The remaining 24 rats were allocated into a model group, a berberine group, and a Coptidis Rhizoma group, each consisting of 8 rats. With the exception of the control group, all rats were provided with 120 µg/mL MNNG solution for free consumption, fed a diet containing 0.05% ranitidine, and administered 0.05 mL/kg of 2% salicylic acid solution by gavage every Tuesday and Friday. 2 mice from each experimental group were randomly chosen for pathological testing after a 26-week period. In the berberine group, six rats were orally administered a daily dose of 50 mg/(kg·d) of berberine solution for 8 weeks. The Coptidis Rhizoma decoction group, consisting of 6 mice, received a daily dose of 2 g/(kg·d) of Coptidis Rhizoma decoction for 8 consecutive weeks. The model group, also comprising six mice, was administered an equal volume of physiological saline by gavage once a day for 8 weeks.

#### Quantitative real-time PCR

Extract mRNA using the Trizol method. According to the instructions of the reagent kit, qRT-PCR was used to detect the mRNA expression of AKT, p-AKT, HIF1a, and VEGF in gastric mucosal tissue, with GAPDH as the internal reference gene.

#### Western blot analysis

Gastric mucosal tissue samples were collected from each group of rats and treated with protein lysis solution. The resulting supernatant was obtained by centrifugation at 12,000 rpm for 30 min and stained using the Coomassie brilliant blue method. Protein content was measured using the BCA method. The supernatant was then combined with sample buffer, denatured, and stored at − 80 °C. Protein blotting was performed to detect various proteins (AKT, p-AKT, HIF-1α, VEGF). Specific Methods: 15 μL of protein samples containing 40 μg were subjected to electrophoresis on a 10% denatured polyacrylamide gel under constant pressure. The proteins were then transferred to a PVDF membrane using constant flow wet transfer, followed by blocking with 5% skimmed milk for 2 h. The membrane was incubated with the corresponding primary antibody at 4 °C for 12 h, then incubated with an alkaline phosphatase-coupled secondary antibody at room temperature for 60 min and developed. GAPDH was used as a primary antibody control. The protein bands were scanned and imaged using a chemiluminescence gel imaging system, and the Lab software was used for band analysis and protein expression evaluation.

#### Statistical analysis

Data analysis was conducted using GraphPad Prism 8 software, with mean ± standard deviation representing all data. Multiple sets of data were compared using one-way ANOVA, with P < 0.05 indicating statistically significant differences.

## Results

### Active ingredient screening of Coptidis Rhizoma

Using the TCMSP database, 48 compounds of Coptidis Rhizoma were obtained, and OB ≥ 30% and DL ≥ 0.18 were used as screening criteria to eliminate active ingredients without corresponding targets, and 11 eligible results were obtained (Table 1, in which we present the 2D structural diagrams of 11 compounds. Compounds CR1 through CR11, respectively).

**Table 1:** The 11 active compounds of Coptidis Rhizoma

ID	Name	2D structure	Database
CR1	Berberine		TCMSP
CR2	Berberrubine		TCMSP
CR3	Berlambine		TCMSP
CR4	(*R*)-Canadine		TCMSP
CR5	coptisine		TCMSP
CR6	Corchoroside A_qt		TCMSP
CR7	epiberberine		TCMSP
CR8	Magnograndiolide		TCMSP
CR9	Palmatine		TCMSP
CR10	Quercetin		TCMSP
CR11	Worenine		TCMSP

### Potential targets screening

**Fig. 2:**
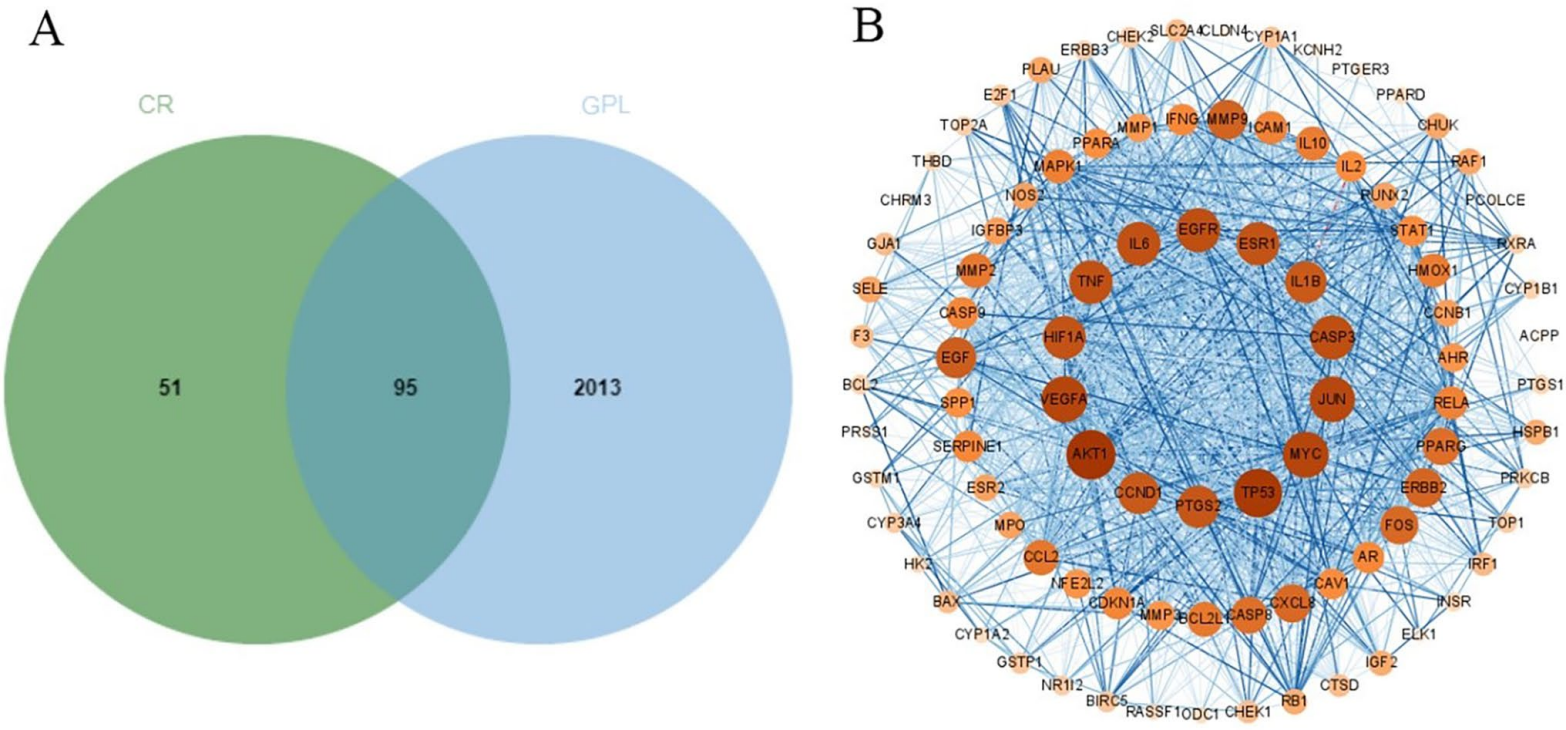
**A** The 95 matched targets common between the predicted Coptidis Rhizoma targets and the gastric precancerous lesions targets. **B** The PPI network. Different colors represent the degree. Node size is proportional to the degree of interaction

The TCMSP database was searched for 11 active ingredients of interest, and 146 targets of TCM were summarised. GPL-related targets were obtained from the GeneCards database and OMIM database, 1588 and 566, respectively, and a total of 2108 targets were obtained by removing duplicates after pooling. By comparing the drug target set and GPL disease target set, 95 potential targets of Coptidis Rhizoma were finally obtained (Fig. 2A).

### PPI network and key module construction

**Fig. 3:**
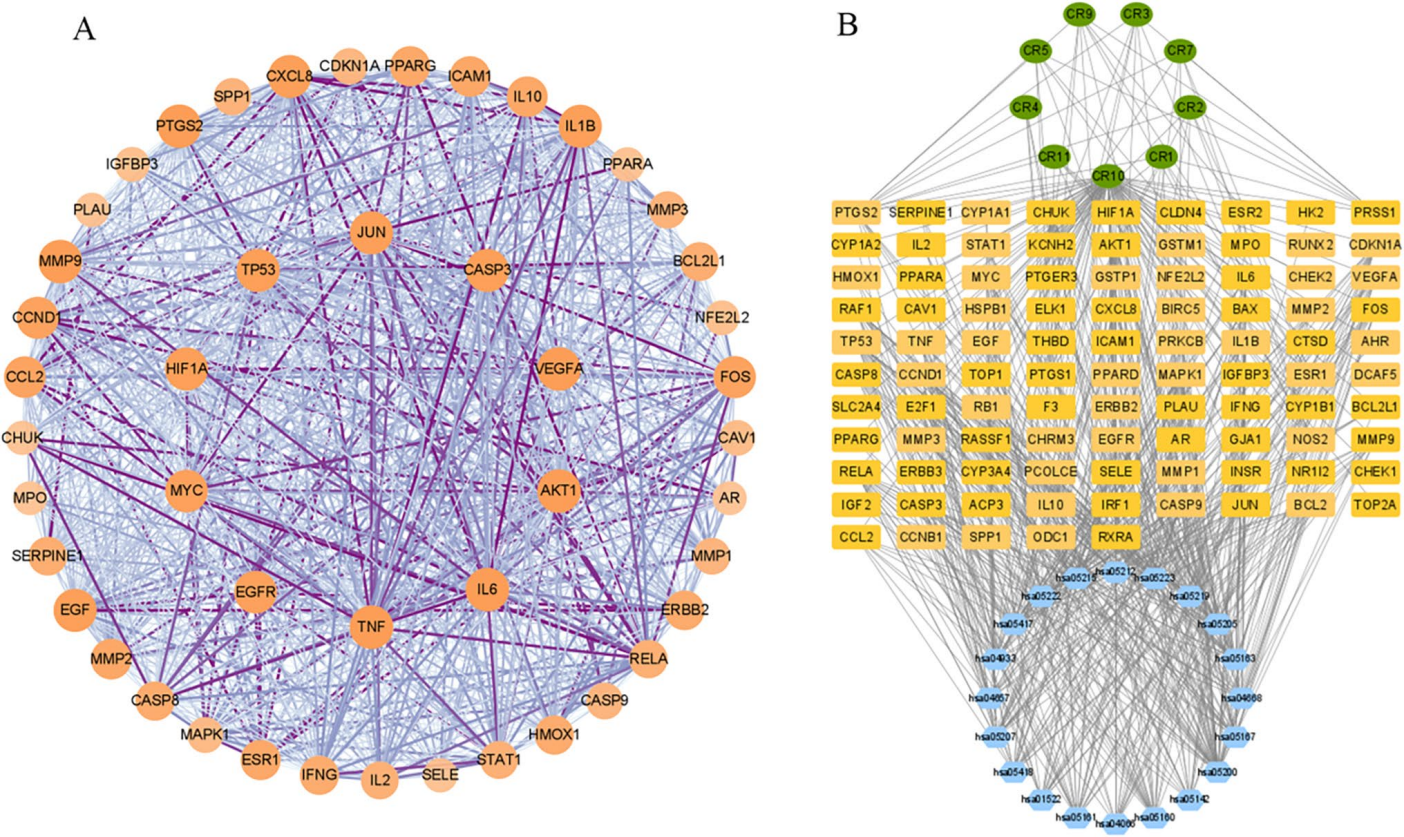
**A** Key module of the PPI network. Different colors represent the degree. Node size is proportional to the degree of interaction. **B** The compound–target–pathway network diagram of Coptidis Rhizomain treating Gastric Precancerous Lesions. The green nodes represent the active compound of Rhizoma Coptidis, whereas the orange nodes represent the anti-gastric precancerous lesions targets of the active compounds, and the blue nodes represent the signalling pathways of the targets. The edges represent the interactions between compounds, targets and pathways

The potential targets obtained from the above screening were uploaded to the STRING database (https://string-db.org/) to obtain PPI network data information. Then, Cytoscape 3.8.2 software was used to construct the PPI network diagram, and a network of 94 nodes and 1593 edges was obtained (Fig. 2B). The PPI network was topologically analysed, and the top 10 core targets were screened with betweenness centrality (BC) greater than 0.00219 (median), closeness centrality (CC) greater than 0.59615 (median), and degree greater than twice the median 62 (Table 2). Key module screening of the network using the MCODE plug-in resulted in a network of 47 nodes with 882 edges (Fig. 3A).

**Table 2:** The top 10 core targets

Gene	Targets	BC	CC	Degree
AKT1	AKT serine/threonine kinase 1	0.04266	0.86111	78
TP53	Tumor protein p53	0.03826	0.84545	76
MYC	MYC proto-oncogene, bHLH transcription factor	0.03211	0.80869	71
VEGFA	Vascular endothelial growth factor A	0.02884	0.80869	71
JUN	Jun proto-oncogene, AP-1 transcription factor subunit	0.03174	0.80172	70
EGFR	Epidermal growth factor receptor	0.03451	0.78151	67
CASP3	Caspase 3	0.02045	0.78151	67
TNF	Tumor necrosis factor	0.04298	0.7750	66
IL6	Interleukin 6	0.02339	0.7750	66
HIF1A	Hypoxia inducible factor 1 subunit alpha	0.01535	0.76859	65

### GO biological process and KEGG pathway enrichment analysis

**Fig. 4:**
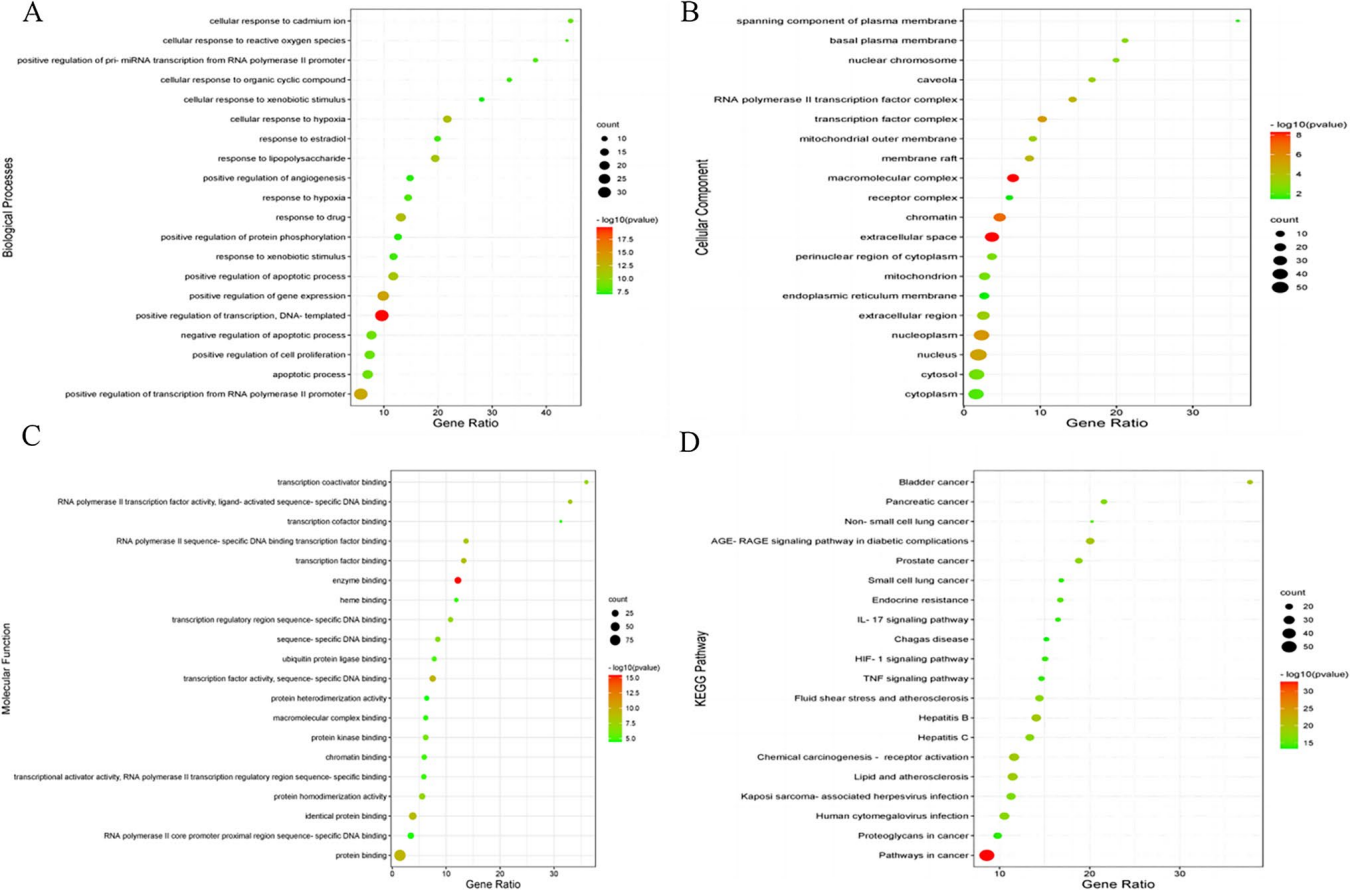
GO biological process and KEGG pathway enrichment analysis of the anti-gastric precancerous lesion targets of Rhizoma Coptidis. **A** Biological processes, **B** cellular component, **C** molecular function, and **D** KEGG pathway

Bioinformatics analysis of the above potential targets using David 6. 8 databases yielded a total of 737 GO terms, including 579 biological processes (BP), mainly involving positive regulation of transcription, DNA-templated regulation, positive regulation of gene expression, positive regulation of transcription from RNA polymerase II promoter, response to hypoxia, cellular response to hypoxia; 54 cellular components (CC), mainly involving macromolecular complex, extracellular space, chromatin, nucleoplasm, transcription factor complex and other aspects; 104 molecular functions (MF), mainly involving binding enzyme, transcription factor activity, sequence-specific DNA binding, protein binding, identical protein binding, and transcription factor binding. According to P < 0.01, the top 20 ranked terms were taken separately for visual plotting (Fig. 4A–C). The above analysis suggested that these potential targets of Coptidis Rhizoma were closely related to the regulation of cellular gene transcription and expression.

A total of 155 pathways were obtained from KEGG signalling pathway enrichment. According to P < 0.01, the top 20 ranked terms were taken for visual plotting (Fig. 4D). Pathways were mainly involved in pathways in cancer, AGE-RAGE signalling pathway in diabetic complications, bladder cancer, hepatitis B, chemical carcinogenesis—receptor activation and so on.

### Build component-target-pathway network diagram

The active ingredients, relevant targets and pathways obtained from the above screening were sorted out to obtain the data information corresponding to each other and imported into Cytoscape 3.8.2 software to generate the “component–target–pathway” network diagram, resulting in a network of 124 nodes and 592 edges (Fig. 3B). Network analysis showed that nine of the 11 active ingredients of Coptidis Rhizoma were involved, of which quercetin (CR10, degree = 90), palmatine (CR9, degree = 9), berberine (CR1, degree = 8), berrubine (CR2, degree = 8), and berlambine (CR3, degree = 8) had high connectivity in the network. In addition, the highest connectivity among the targets was RAC-alpha serine/threonine-protein kinase (AKT1), degree = 19), Mitogen-activated protein kinase 1 (MAPK1, degree = 19), and the highest connectivity in KEGG pathways were Pathways in cancer (hsa05200, degree = 50), lipid and atherosclerosis (hsa05417, degree = 27), and chemical carcinogenesis- receptor (hsa05207, degree = 27). Accordingly, we conclude that the main active components of Coptidis Rhizoma, such as quercetin and berberine may intervene in the occurrence and progression of gastric cancer lesions by inhibiting tumor signalling pathways.

### Molecular binding capacity validation

**Fig. 5:**
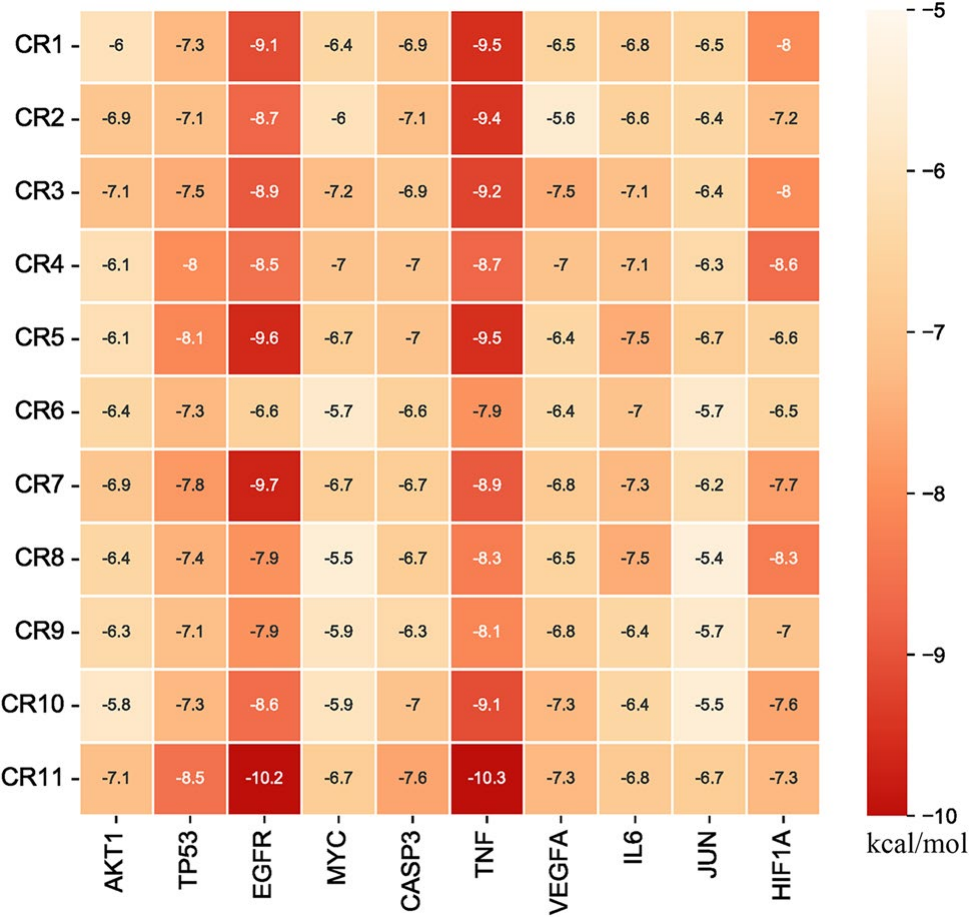
The heat map of binding energy between active components of Coptidis Rhizoma and the core targets

**Fig. 6:**
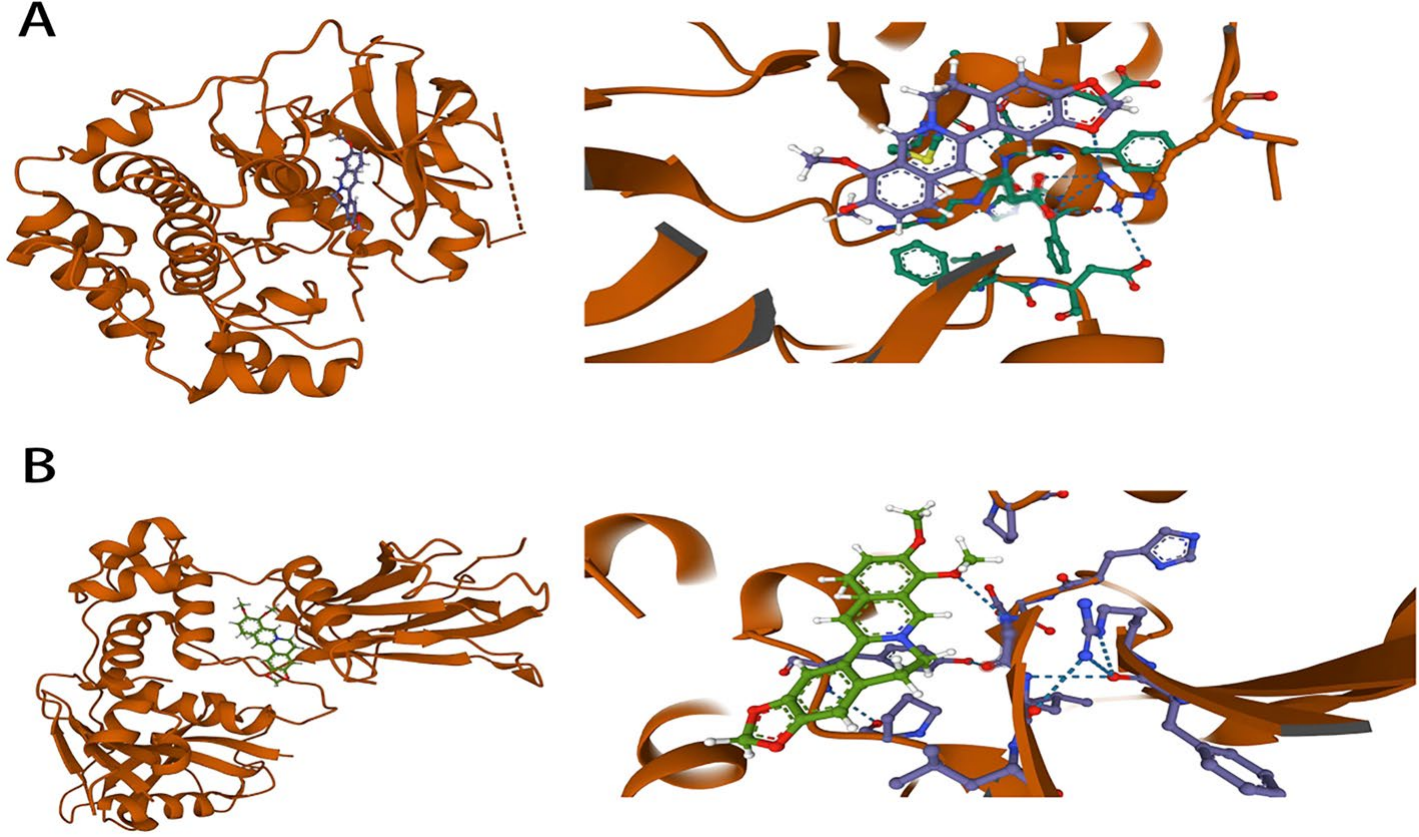
Schematic 3D representation of the molecular docking model. Binding mode of berberine (CR1) to AKT (**A**), berberine (CR1) to HIF-1α (**B**)

Molecular docking techniques were used to verify intermolecular binding capacity. The top 10 selected core targets above were used as receptors for molecular docking with 11 active ingredients. Core target protein structure files (pdb) were obtained from the PDB database, and active ingredient molecular structure files (mol2) were obtained from the TCMSP database. Auto Dock Tools 1.5.7 software was used to perform pre-docking processing of receptor files, including dehydrating molecules, hydrogenation, calculating the total charge, and setting Grid Box parameters, which were saved in “pdbqt” format. The selected ligands and receptors were subjected to batch molecular docking one by one using AutoDock Vina 1.2.3 software, and the results showed that each target had a good binding ability to the active components, and the Vina affinity values were less than − 5.0 kcal/mol (Fig. 5). The key compound–target interactions were also performed using PyMoL Version 2.4.0 (Fig. 6).

### Berberine and Coptidis Rhizoma decoction inhibit AKT/HIF-1α/VEGF signaling pathway

**Fig.7:**
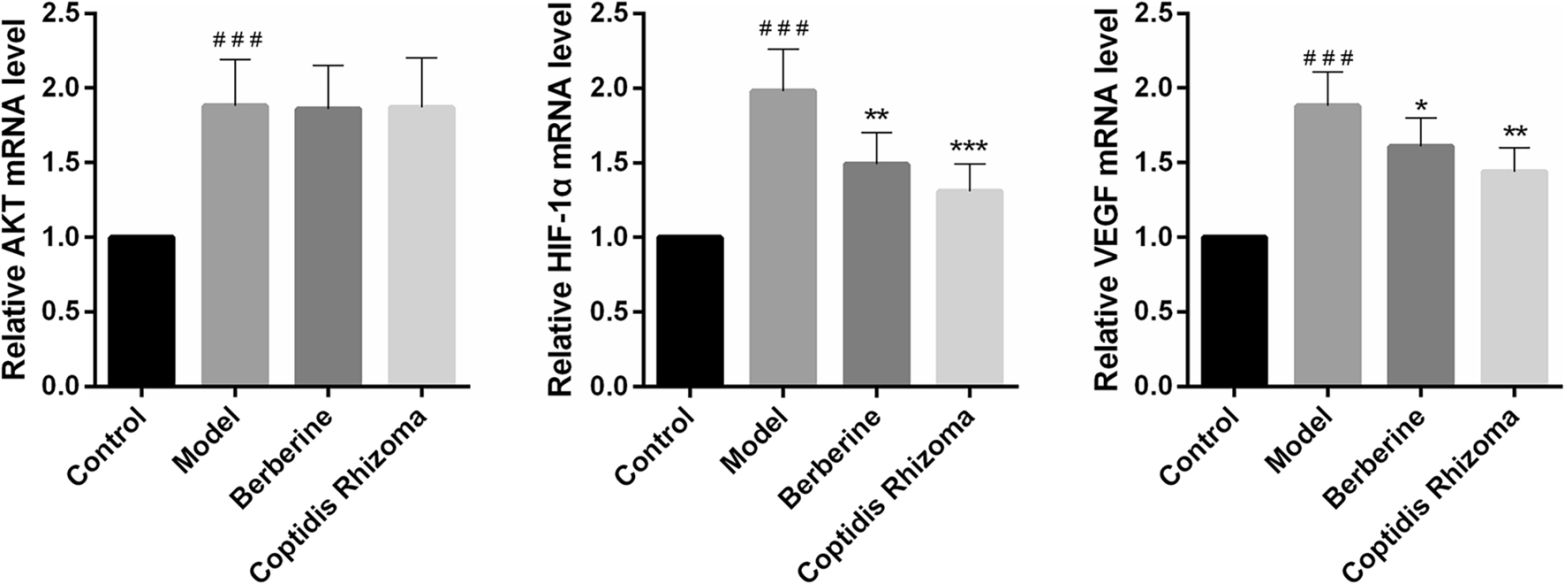
The mRNA levels of AKT, HIF-1α and VEGF. ^###^p < 0.001 compared with Control group, *p < 0.05, **p < 0.01 and ***p < 0.001 compared with Model group

In comparison to the control group, the model group exhibited a statistically significant increase in mRNA expression of VEGF alongside AKT and HIF-1α (P < 0.001). Conversely, the Berberine group and Coptidis Rhizoma decoction group demonstrated a statistically significant decrease in mRNA expression of VEGF and HIF-1α compared to the model group (P < 0.01). These findings suggest that Berberine and Coptidis Rhizoma possess inhibitory effects on HIF-1α and VEGF mRNA expression in rats with gastric precancerous lesions (Fig. 7).

**Fig.8:**
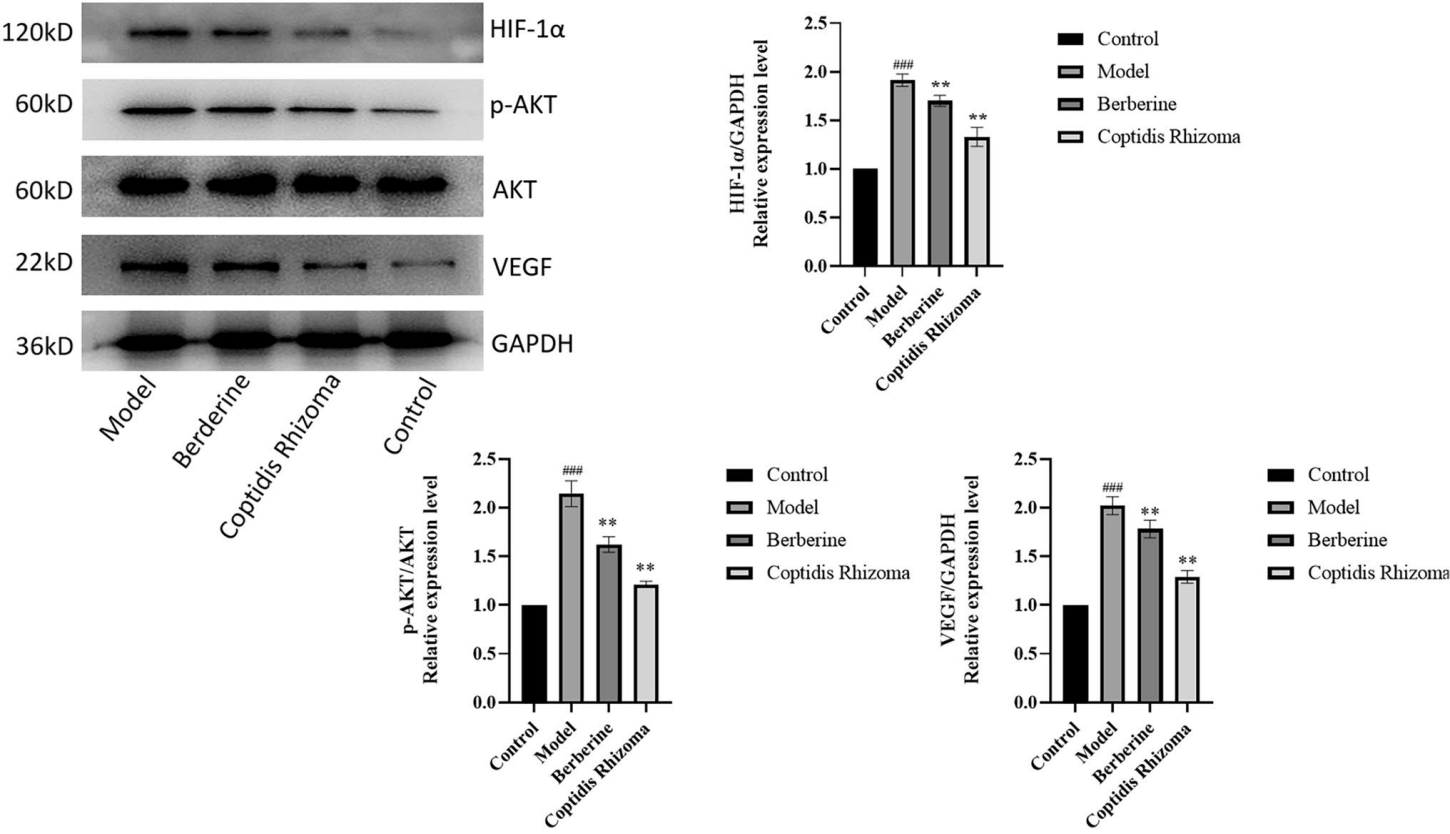
The protein levels of p-AKT, HIF-1α and VEGF. ^###^p < 0.001 compared with Control group, **p < 0.01 compared with Model group

In comparison to the control group, the model group exhibited a significant increase in the expression of AKT and HIF-1α, as well as VEGF protein, with a statistically significant difference (P < 0.001). Conversely, the Berberine group and Coptidis Rhizoma decoction group demonstrated a decrease in p-AKT and HIF-1α levels, as well as VEGF protein, compared to the model group, with a statistically significant difference (P < 0.01). These findings suggest that Berberine and Coptidis Rhizoma possess the ability to inhibit p-AKT and HIF-1α, as well as VEGF protein expression in rats with gastric precancerous lesions (Fig. 8).

## Discussion

TCM is an important part of the world health-care system [25]. It has a unique diagnosis and treatment system—“syndrome differentiation and treatment”, which can diagnose and treat diseases from a macroscopic point of view, especially those difficult lesions with unknown aetiology and molecular mechanisms. Therefore, it is widely used in the prevention and treatment of a series of refractory diseases, including inhibition of tumor proliferation, induction of apoptosis and prevention of complications [26]. The mechanism of GPL is unknown, and there is a lack of effective methods to reverse gastric mucosal lesions, making the treatment of GPL challenging [10]. It is worth mentioning that Chinese medicine has achieved good efficacy in the treatment of GPL [27]. Coptidis Rhizoma, a staple in TCM with a long-standing history of use for gastrointestinal and diabetic conditions, is recognized for its safety in clinical applications, with no observed hepatotoxic or nephrotoxic effects to date. However, due to its inherently “cold” nature according to Traditional Chinese Medicine theory, prolonged use may lead to gastrointestinal discomfort [28, 29]. It is also the mainstay of many Chinese herbal compounds for the treatment of GPL, such as Huazhuojiedu decoction, Qinhuayin, and Weixibaonizhuan pills [27, 30, 31]. In vitro experiments have demonstrated that the main extracts of Coptidis Rhizoma, such as berberine and quercetin, have antitumor activities, such as inhibiting angiogenesis and inducing apoptosis [32–34]. However, its specific mechanism of action has not been fully elucidated. Therefore, we used network pharmacology and molecular docking methods to explore the potential molecular mechanisms of active compounds of Coptidis Rhizoma systematically and comprehensively in the treatment of GPL.

In our study, 11 active ingredients and 146 corresponding targets of Coptidis Rhizoma were first obtained from TCMSP database mining. In addition, 2108 targets of GPL were obtained from the GeneCards database and OMIM database. Comparing the drug target set and GPL disease target set, 95 potential targets of Coptidis Rhizoma were finally obtained. The active components of Coptidis Rhizoma mainly include alkaloids and flavonoids, all of which have good pharmacokinetic properties. According to the network diagram analysis of “component–target–pathway”, alkaloids and flavonoids of Coptidis Rhizoma are the main active components of anti-tumor properties. This is consistent with modern medical confirmation that these alkaloids and flavonoids can inhibit PI3K/AKT signalling pathway and tyrosine kinase, thereby inducing apoptosis and inhibiting tumor proliferation [35–38].

The top five potential targets were AKT1, TP53, VEGFA, MYC and JUN. AKT1 is a member of the serine-threonine protein kinase family that regulates many cellular processes, including metabolism, proliferation, cell survival, growth, and angiogenesis [39]. AKT1 is a key regulator of cell proliferation [40] and drives cancer progression by transducing extracellular signals to the cytoplasm and nucleus through phosphorylation [41]. TP53 gene is a cell cycle-related gene, which plays an important role in cell proliferation and apoptosis by regulating cell cycle-related protein synthesis. Mutation or deletion of TP53 leads to cell cycle disturbance and apoptosis inhibition [42]. TP53 mutations are often present in premalignant lesions and may precede morphologic changes from adenomas to carcinomas [43, 44]. VEGFA is a key pro-angiogenic ligand, and highly expressed VEGFA often stimulates angiogenesis and growth and is involved in tumor angiogenesis and metastasis [45, 46]. MYC is a well-established oncogene involved in cell cycle progression, apoptosis, and cell transformation [47]. Important interactions exist between MYC and TP53 in many different cancers [48]. MYC amplification and TP53 mutations have synergistic effects in promoting histological grading phenotypes from low-grade tumors to high-grade tumors [49]. Activation of JUN and Fos phosphorylation leads to the formation of AP-1 complexes that activate the transcription of target genes and regulate angiogenesis-related cell proliferation, differentiation, survival, and migration [50]. In conclusion, mutations such as AKT1, TP53, VEGFA, MYC, and JUN are likely to have been present in gastric precancerous lesions, showing an imbalance between cell proliferation and apoptosis. The active components of Coptidis Rhizoma may regulate malignant progression, such as cell proliferation and apoptosis, angiogenesis and growth, by binding to the above-related receptors.

GO enrichment analysis showed that the mechanism of Coptidis Rhizoma acting on GPL involved multiple biological processes and molecular functions, of which the most relevant biological processes were gene expression regulation and apoptosis regulation, and the most relevant molecular functions were enzyme binding, transcription factor activity, sequence-specific DNA binding, and protein binding (enzyme binding, transcription factor binding activity, sequence-specific DNA binding, protein binding). From KEGG signalling pathway analysis, GPL are most closely related to two signalling pathways, the cancer pathway and the AGE-RAGE signalling pathway in diabetic complications. It is easy to understand that abnormal cancer signalling pathways underlie the development of GPL. Diabetic complications AGE-RAGE signalling pathway is associated with a series of complications of diabetes, such as retinopathy, neuropathy, cardiovascular lesions, and so on [51–54], which need not be repeated here. AGE-RAGE signal axis was also strongly correlated with GPL. It has been shown that AGE-RAGE intracellular signal transduction usually involves PI3K/Akt signalling pathway and MAPK/ERK pathway and plays a key role in regulating cell proliferation and cell differentiation [55, 56]. At the same time, the AGE-RAGE signalling axis is also involved in VEGF-mediated angiogenesis, and cellular experiments have confirmed that RAGE receptors are positively associated with increased vascular density and cancer progression [57, 58]. This also demonstrates from the side that the occurrence of tumors has an important link with diabetes [59].

**Fig. 9:**
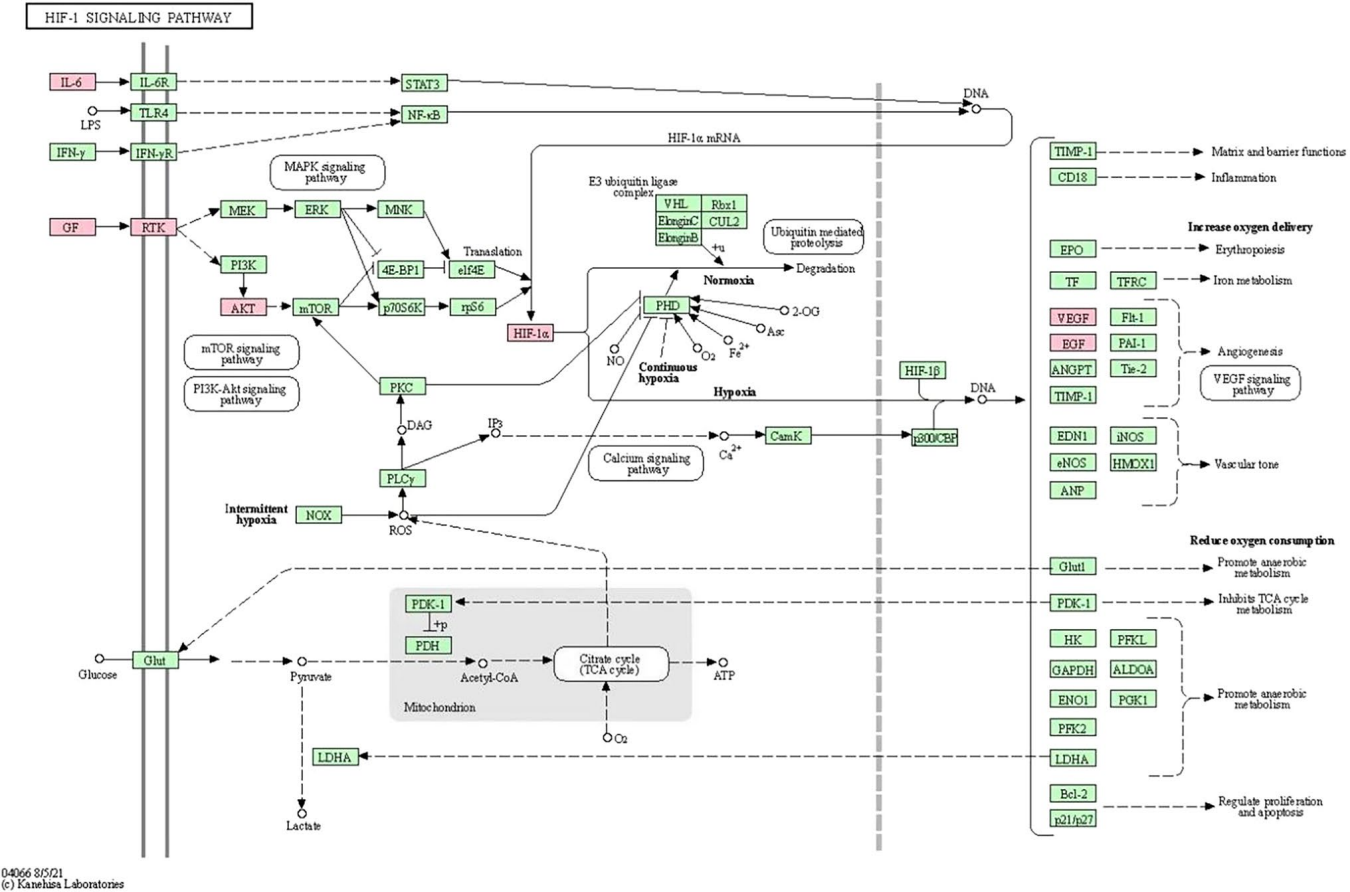
HIF-1α pathway performed by KEGG mapper (ID: hsa04066); the putative targets and the genes implicated in the pathway are shown in pink

As with tumor development, precancerous lesions are a complex disease process that may involve the dysregulation of multiple oncogenic signalling pathways (pathways in cancer). Seven of the top 20 genes exhibit enrichment in the HIF-1α signal pathway (Fig. 9). EGFR is located on the cell membrane and is an upstream part of the PI3K/AKT signalling pathway. Epidermal growth factor receptor (EGFR) is a cell surface receptor tyrosine kinase (RTK) with intrinsic protein tyrosine kinase activity [60, 61]. EGFR is auto phosphorylated by tyrosine kinases when growth factors bind to EGFR [62], subsequently activating the downstream PI3K/AKT pathway [63]. This process plays a crucial role in gastric mucosal repair, regulation of mucosal cell growth and development, repair of damaged mucosa, and modulation of differentiation, proliferation, and maturation of gastrointestinal epithelial cells [64, 65]. Additionally, AKT has the capacity to facilitate HIF-1α signal pathways, thereby enhancing the expression of VEGF and subsequently causing dysregulation in cell metabolism, growth, and differentiation [66–68]. The molecular docking analysis indicates that the active compounds found in Coptidis Rhizoma exhibit strong binding affinity with core of targets. Therefore, we speculate that these compounds may potentially modulate the AKT/HIF-1α/VEGF signalling pathway to prevent excessive differentiation of gastric mucosal cells, thereby reducing the risk of atrophy and dysplasia. In an experimental rat model, the levels of AKT and HIF-1α, as well as VEGF, were significantly elevated in the control group, but were effectively suppressed by treatment with either berberine or Coptidis Rhizoma decoction. This provides evidence supporting the therapeutic efficacy of Coptidis Rhizoma.

## Conclusion

TCM plays an irreplaceable role in treating difficult lesions with unknown aetiology and molecular mechanisms. According to the theory of TCM, GPL manifest as gastric mucosal atrophy with or without IM, characterized by a tendency to become cancerous and attributed to internal dampness. Coptidis Rhizoma can clear away heat and dampness, purge fire and detoxify, with the effect of anti-GPL. We used network pharmacology and molecular docking methods to investigate its active components, potential key targets of action, and related signalling pathways. This study has demonstrated that the primary bioactive compounds found in Coptidis Rhizoma, including Berberine, Berberrubine, and Berlambine, possess the ability to suppress AKT and HIF-1α, thereby modulating the expression of VEGF to govern the differentiation, proliferation, and maturation of gastric epithelial cells. This process facilitates the restoration of gastric mucosa, ultimately reversing atrophy and potentially intestinal metaplasia. The mechanism underlying this regulatory effect predominantly involves the inhibition of HIF-1α through signaling pathways. However, this study acknowledges two principal limitations. First, there is an inherent uncertainty regarding the extent to which the animal model can accurately replicate the complexities of human gastric precancerous lesions. Second, the translational applicability of findings from animal studies to the human context may be fraught with potential discrepancies. Moreover, the research may not fully encompass the intricate network of signaling pathways implicated in the development of gastric precancerous lesions. This suggests that there is a necessity for further in-depth investigation, particularly focusing on the molecular and cellular dimensions.

### Supplementary Information

Below is the link to the electronic supplementary material.

Supplementary file 1 (PDF 1144 KB)

Supplementary file 2 (PDF 1145 KB)

## Data Availability

The data that support the findings of this study are available from the corresponding author, [Xiaobo Wang], upon reasonable request.
