# Supplementary figures and images for: Exploring the therapeutic mechanisms of Coptidis Rhizoma in gastric precancerous lesions: a network pharmacology approach
Source: Discov Oncol. 2024 Jun 5;15:211. doi: 10.1007/s12672-024-01070-5 (PMC11153449; doi:10.1007/s12672-024-01070-5)

1. HIF1-A

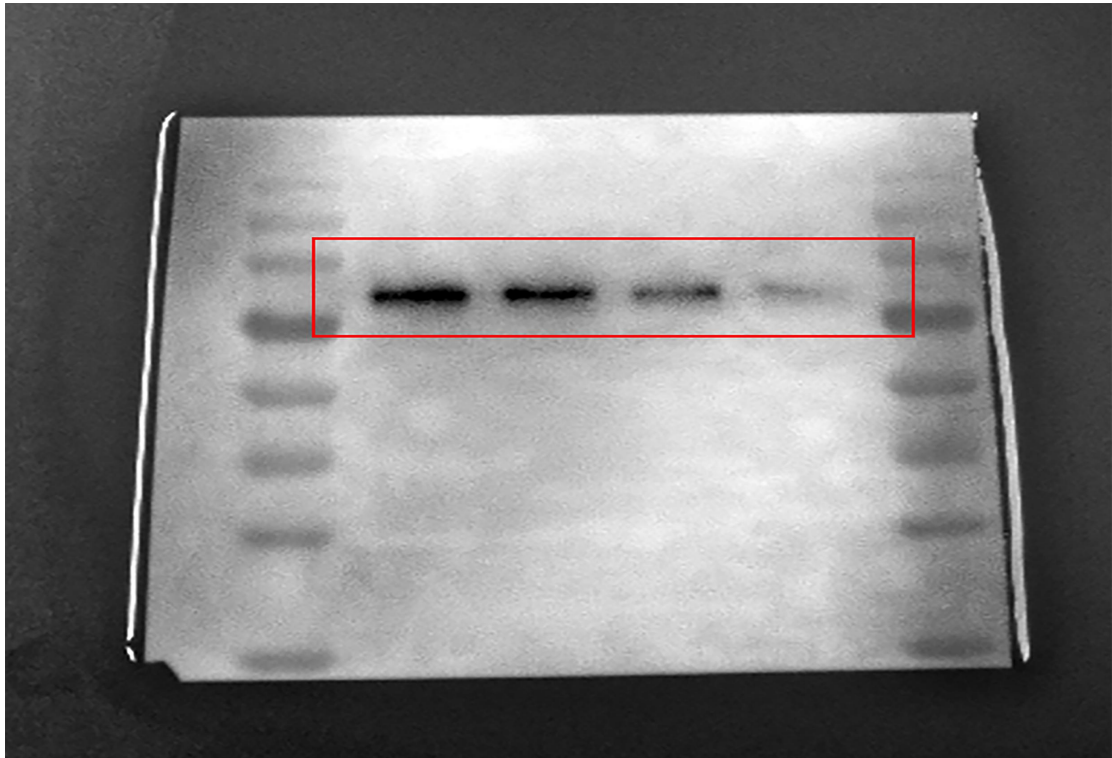

2. p-AKT

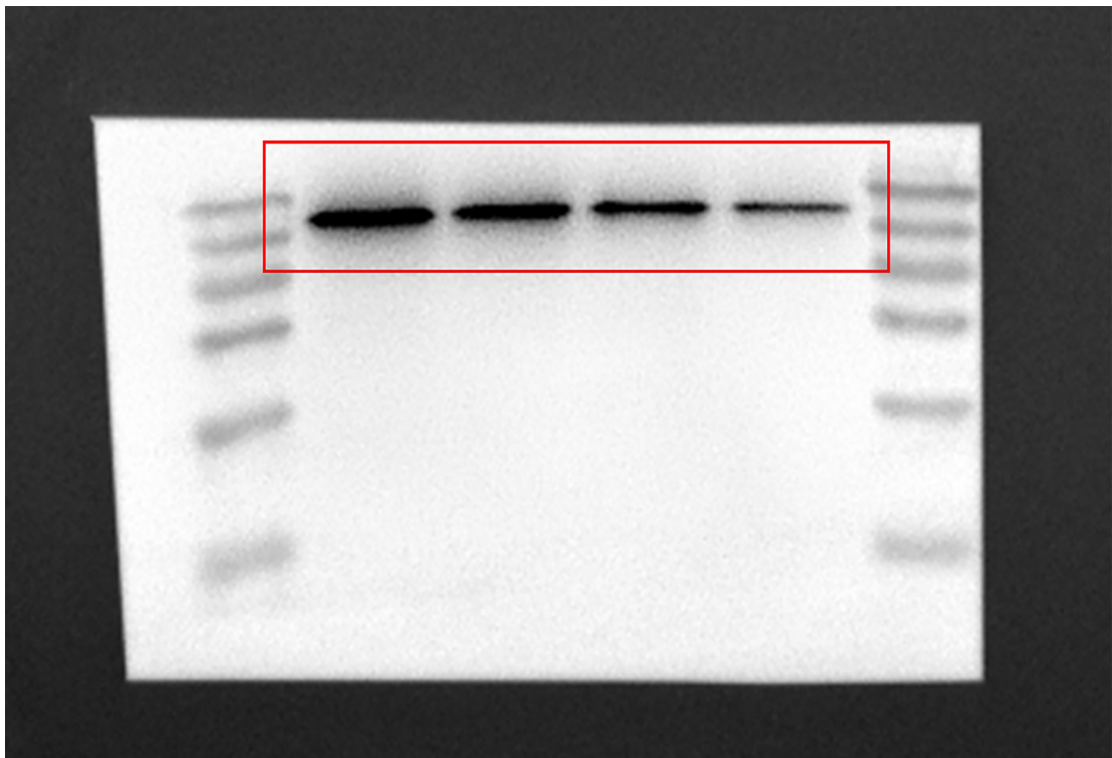

3.AKT

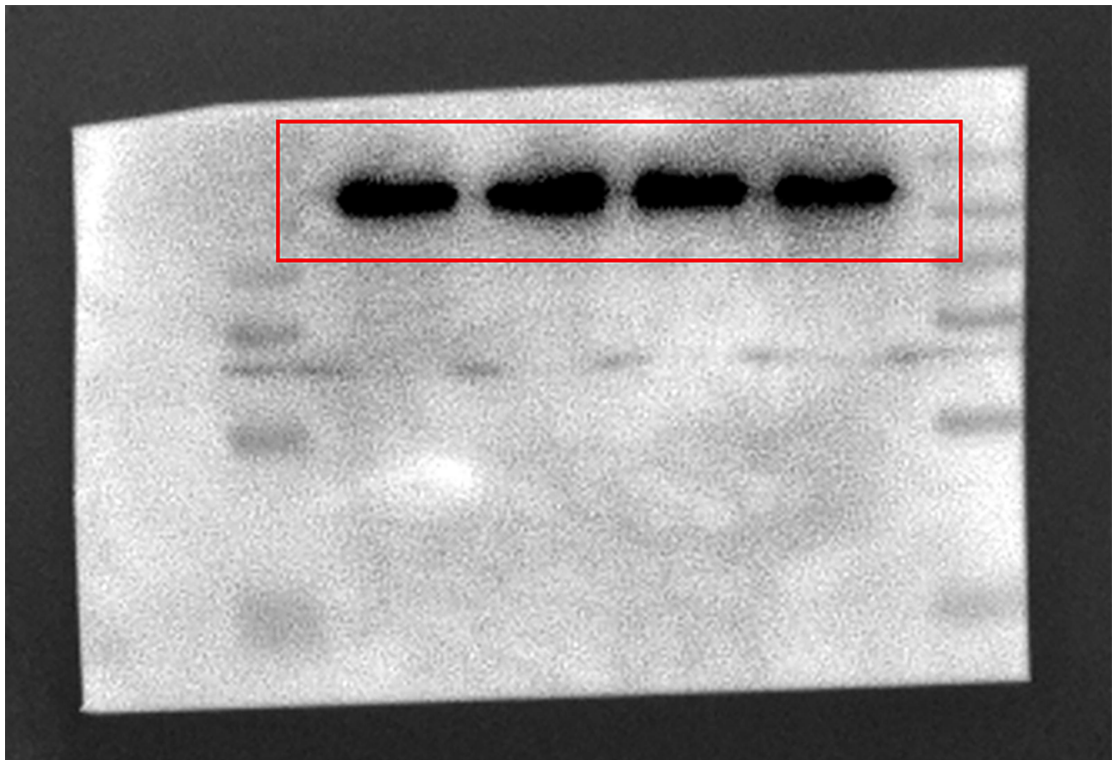

4.VEGF

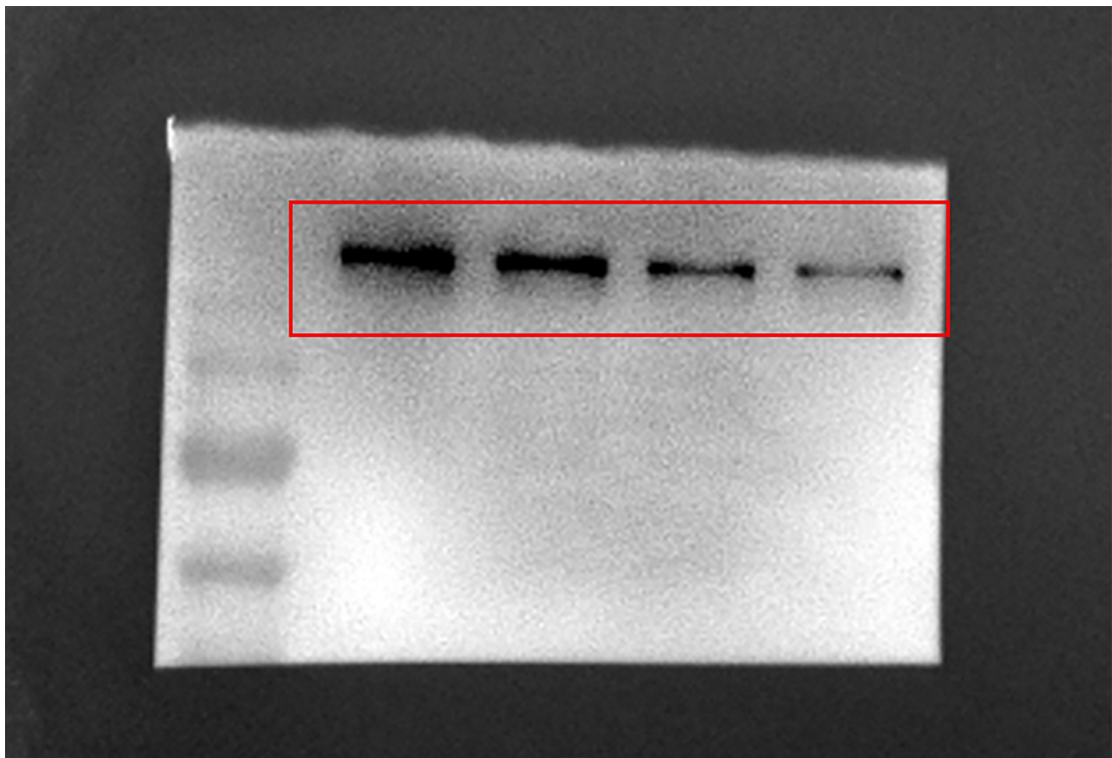

## 5.GAPDH

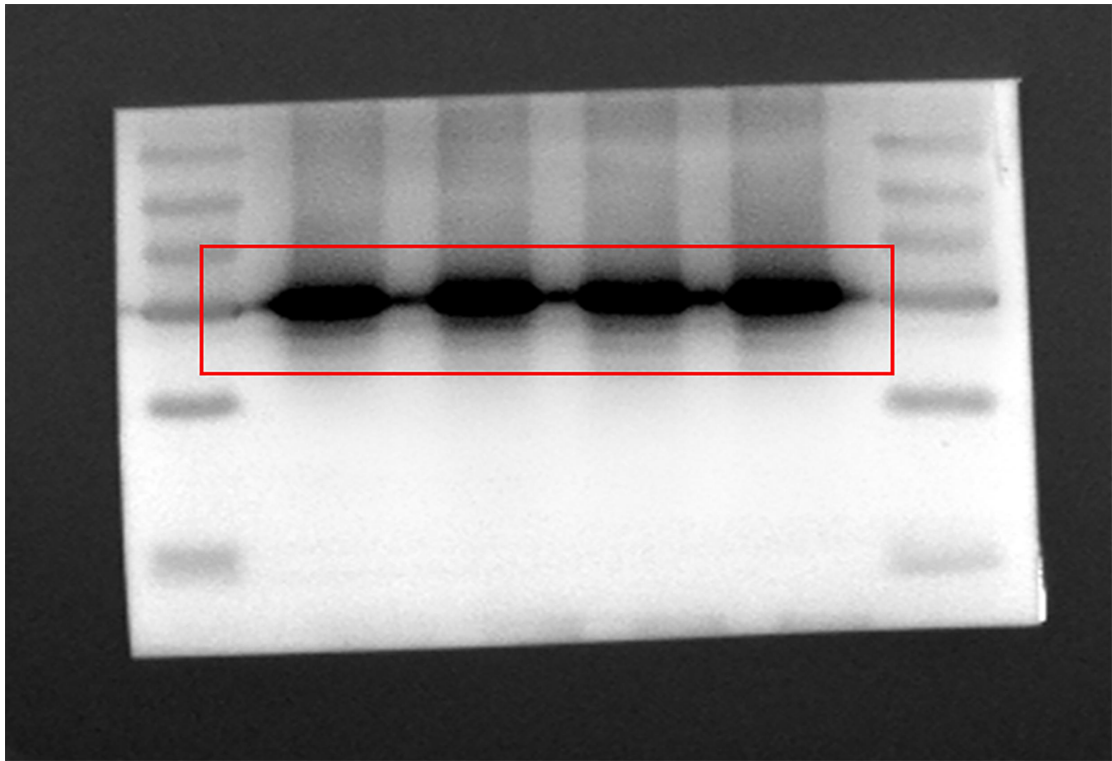

Supplement: Supplementary file 1 — (PDF 1144 KB) [file 12672_2024_1070_MOESM1_ESM.pdf]

## 1.HIF-1A

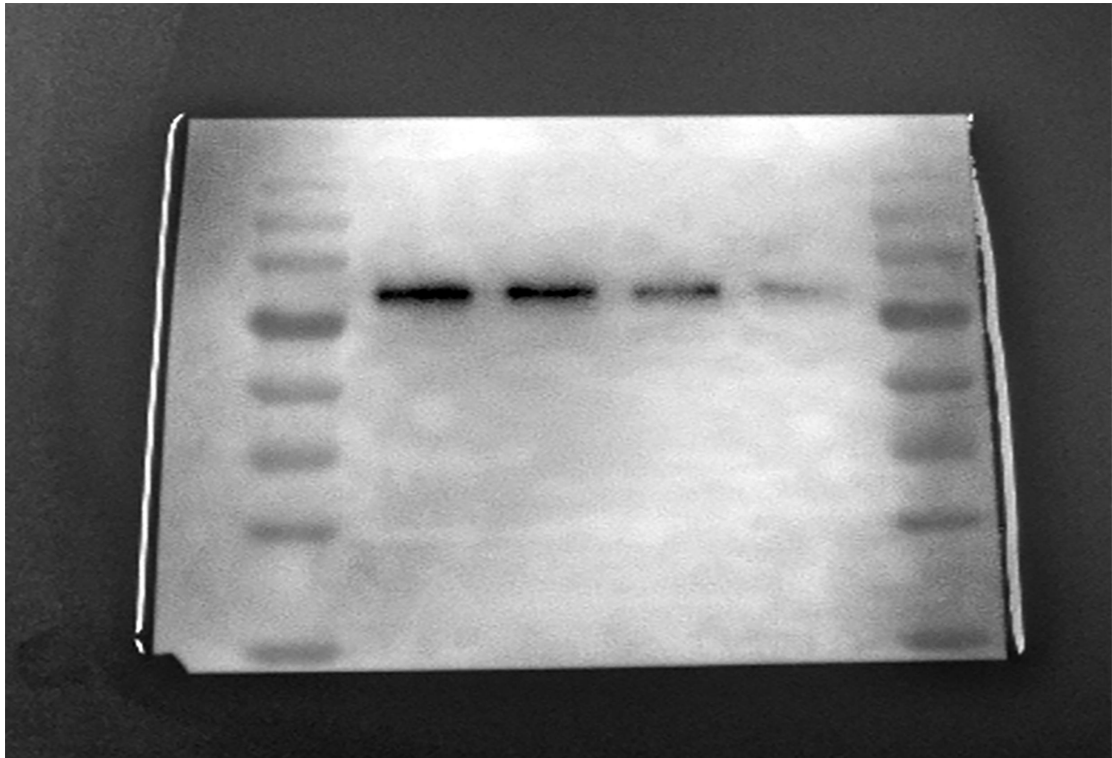

## 2.p-AKT

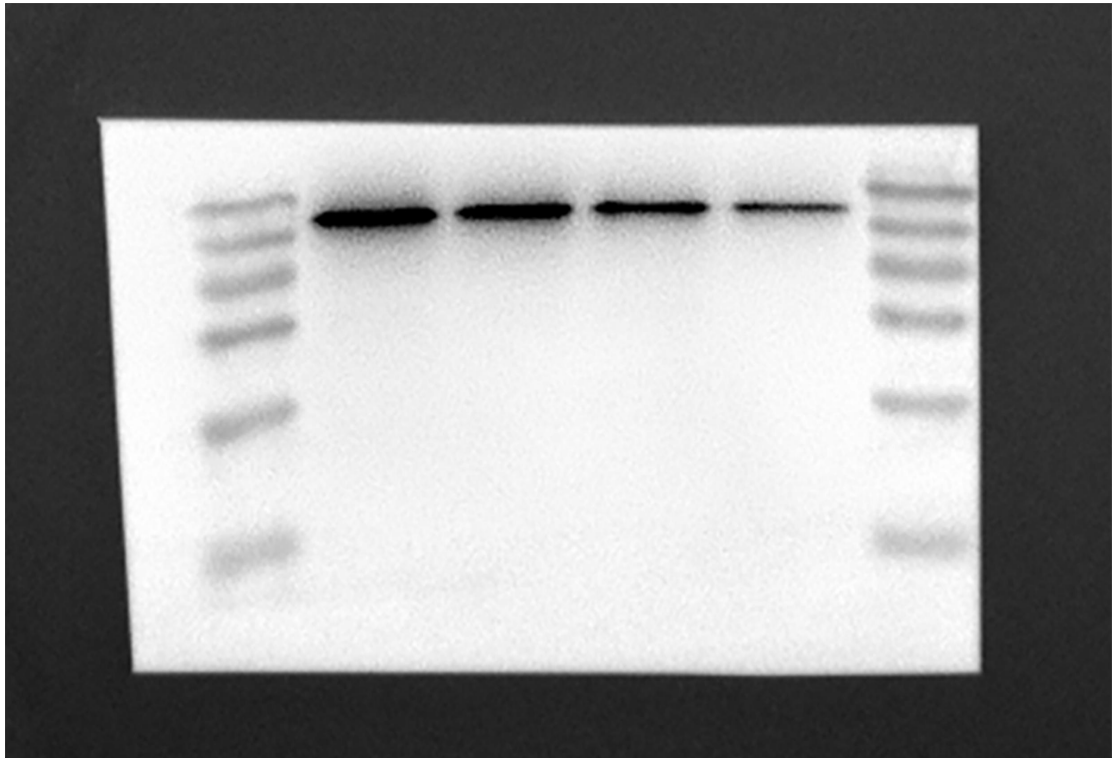

3.AKT

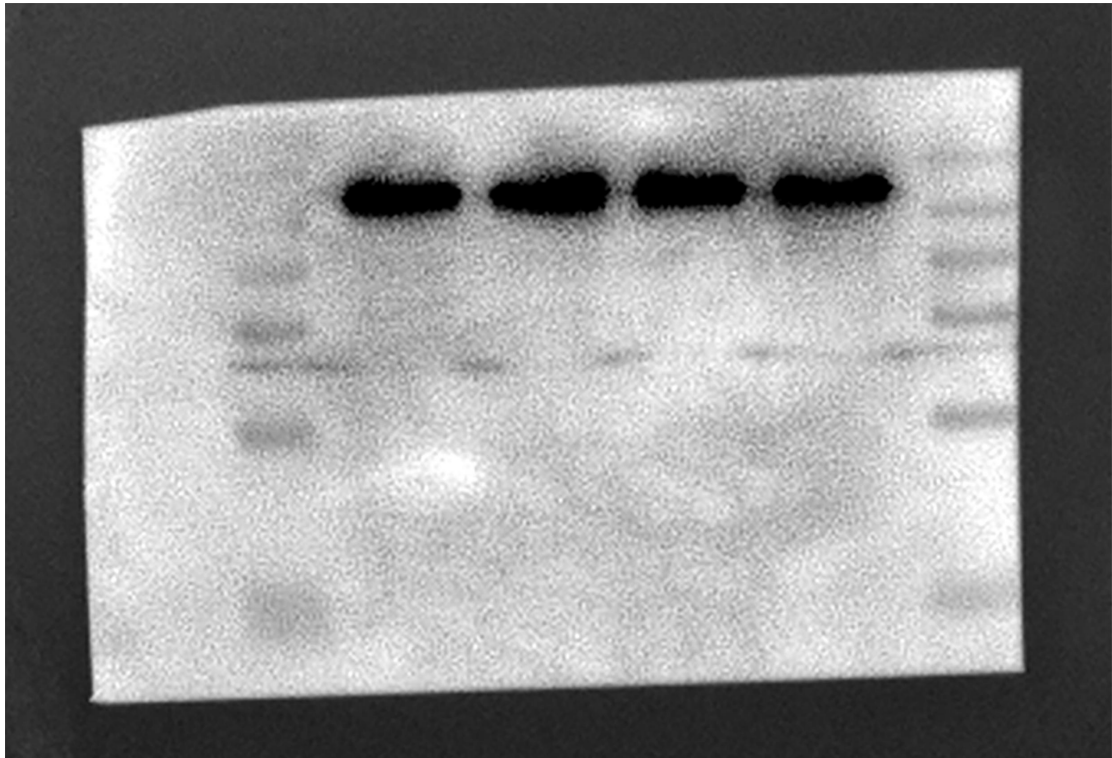

4.VEGF

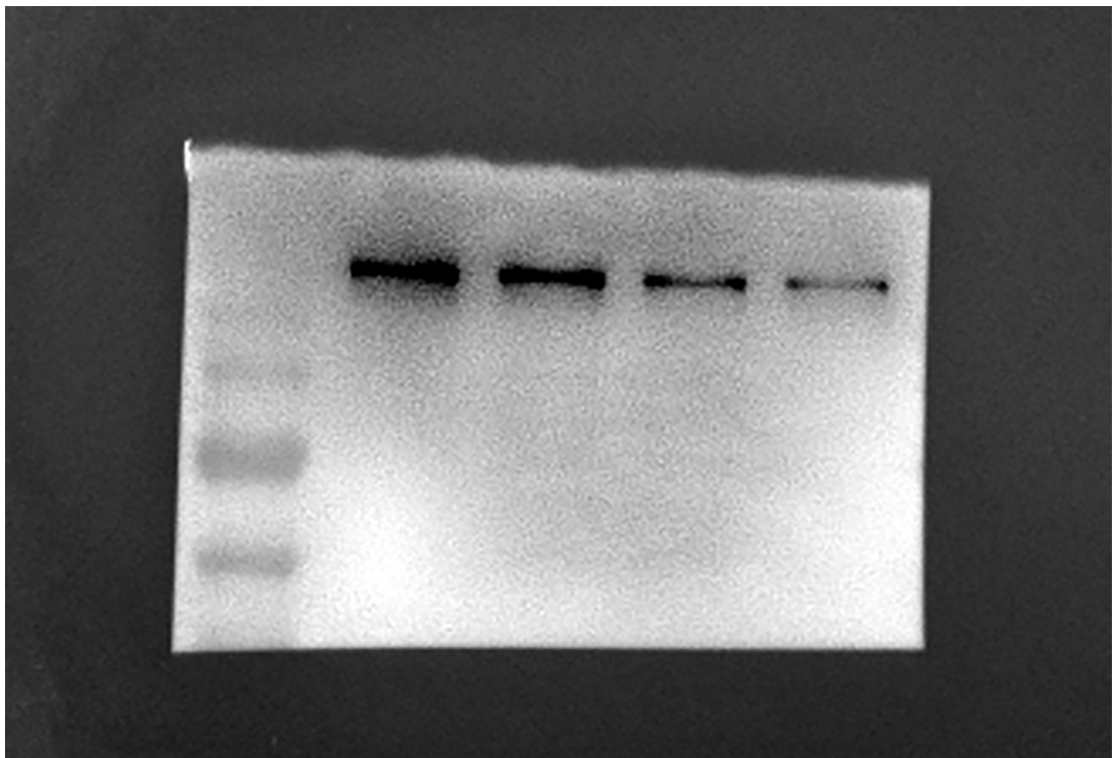

## 5.GAPDH

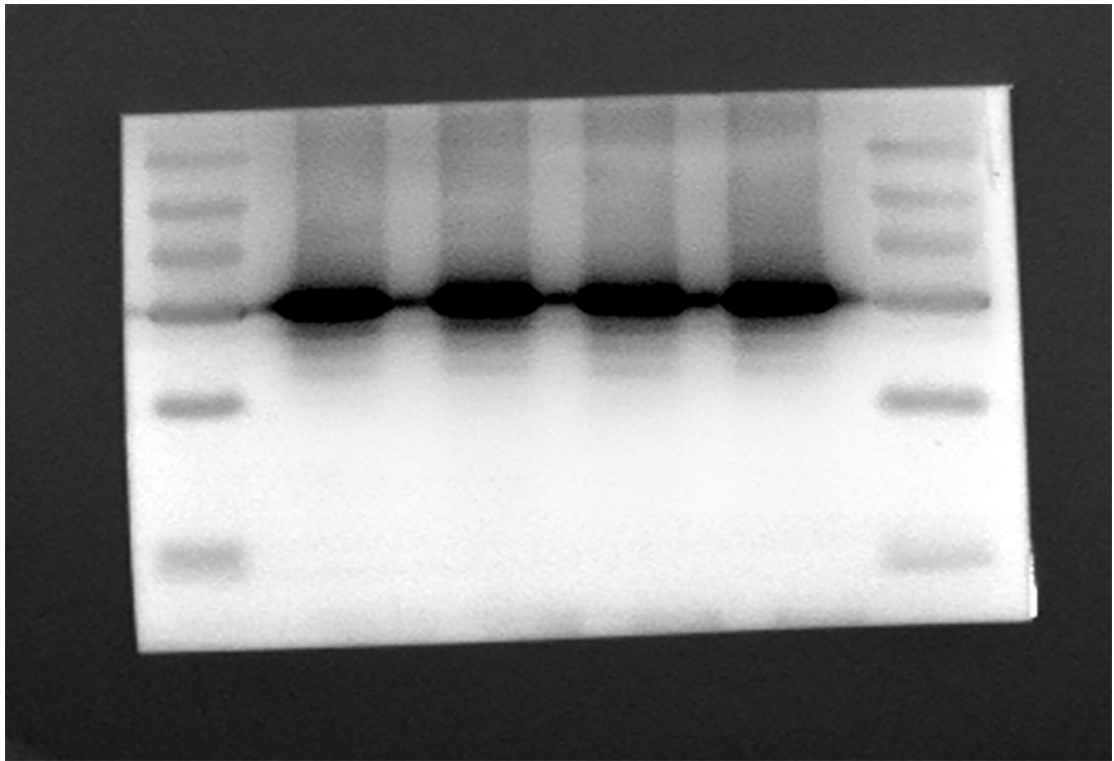

Supplement: Supplementary file 2 — (PDF 1145 KB) [file 12672_2024_1070_MOESM2_ESM.pdf]
